# Urothelial carcinoma with an *NRF1-BRAF* rearrangement and response to targeted therapy

**DOI:** 10.1101/mcs.a003848

**Published:** 2019-06

**Authors:** Alexandra L. Isaacson, Natalya V. Guseva, Aaron D. Bossler, Deqin Ma

**Affiliations:** Department of Pathology, University of Iowa Hospitals and Clinics, Iowa City, Iowa 52242, USA

**Keywords:** neoplasm of the ureter, renal pelvic carcinoma

## Abstract

Although *BRAF* mutations are commonly identified in many solid tumors and the response of *BRAF* p.V600E-positive tumors to targeted therapy is well documented, *BRAF* rearrangements are less frequent and are predominantly found in low-grade glioma, melanoma, lung, colorectal, and thyroid carcinoma. Preclinical and clinical studies have demonstrated effectiveness of multiple therapies (RAF-targeted, ERK-targeted, or MEK-targeted) targeting *BRAF*-fusion harboring tumors. We report a rare *NRF1-BRAF* fusion with novel breakpoints, identified by next-generation sequencing–based assay, from a 69-year-old man with metastatic urothelial carcinoma (UC) of the renal pelvis and his initial clinical response to a second-generation MEK inhibitor, trametinib, before stopping the medication because of adverse side effects. The *NRF1-BRAF* fusion has only been reported in a single case of anaplastic pleomorphic xanthoastrocytoma, and *BRAF* rearrangement has never been reported in UC.

## CASE PRESENTATION

**Figure 1.**
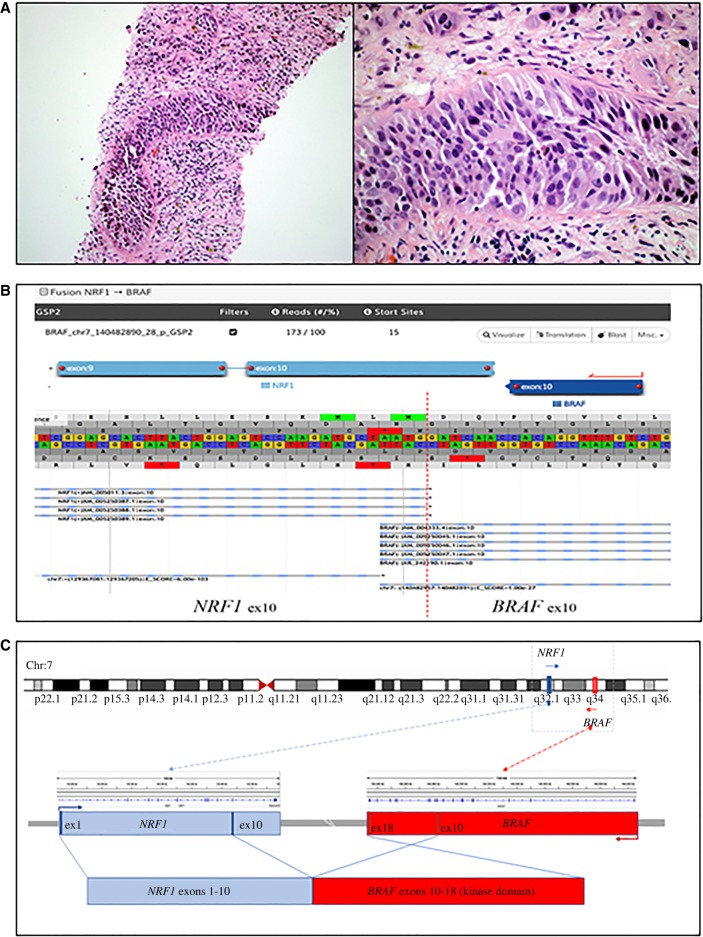
(*A*) H&E slide of metastatic urothelial carcinoma to liver, 4× and 20× view. (*B*) JBrowse view of *NRF1*ex10-*BRAF*ex10 fusion detected by Archer Analysis. Dotted vertical red line indicates the fusion breakpoint. (*C*) Schematics of *NRF1-BRAF* fusion formation. Blue and red represent *NRF1* (NM_005011.5) and *BRAF* (NM_004333.5) transcripts, respectively. (Ex) Exon.

A 69-year-old man with a past medical history of hypertension and coronary artery disease presented with hematuria and a 2.6-cm mass in the left kidney. He underwent left radical nephroureterectomy and was found to have a high-grade papillary urothelial carcinoma (UC) in the renal pelvis with invasion into renal parenchyma and lymph node metastasis (AJCC 8th edition: pT3N1). One month later, a magnetic resonance imaging (MRI) scan demonstrated metastatic disease in his liver, cervical and lumbar spines, humerus, and retroperitoneal lymph nodes. A liver biopsy confirmed the presence of metastatic UC (Fig. 1A). He received eight cycles of carboplatin and gemcitabine that resulted in disappearance of the liver lesion and decrease in size of the bone lesions and lymphadenopathy. He also received denosumab (Xgeva) during this time. He required several blood transfusions for iron deficiency anemia and experienced mild neuropathy as a side effect of chemotherapy.

Two months after completing chemotherapy, an MRI scan showed disease progression in the liver and retroperitoneum. The patient enrolled in a phase II trial of nivolumab (Opdivo), an anti-PD-1 antibody. He tolerated the therapy well, but 2 months later, restaging imaging showed an increase in the size of the liver, retroperitoneum, pelvic, and inguinal lymph node disease. A second biopsy of the liver lesion was evaluated with the FoundationOne test (Foundation Medicine) and at our institution using next-generation sequencing (NGS)-based panels. An *NRF1-BRAF* fusion was detected by both laboratories.

Based on the genomic findings, the patient opted to begin a trial of trametinib (Mekinist), a second-generation mitogen-activated protein kinase kinases (MEK) inhibitor. After two and a half months of treatment, a MRI scan demonstrated an overall 48.4% decrease in size of the liver lesions: from 6.3 cm to 2.4 cm in segment 8; from 6.6 cm to 3.6 cm in segment 5; and from 2.8 cm to 1.6 cm in segment 2. However, at 3 months post-initiation of treatment, the patient's chest computed tomography (CT) scan showed ground-glass opacifications concerning for pneumonitis, a known adverse effect of trametinib. The patient was advised to stop taking his medication for 3 weeks. In the interim period, he developed cyclic fevers, fatigue, and confusion with leukocytosis and elevated liver enzymes. An MRI scan demonstrated new liver lesions suspicious for disease progression, and a repeat liver biopsy confirmed new foci of metastatic UC. Given his poor performance status, the patient opted to enter hospice care and died shortly afterward.

## TECHNICAL ANALYSIS

A formalin-fixed, paraffin-embedded (FFPE) block with tumor was sent to FoundationOne for comprehensive genomic analysis.

### Sample Preparation and Testing at Our Institution

One hematoxylin and eosin (H&E)-stained slide along with 10 unstained sections (6 µm in thickness) were cut from the same block that was tested at FoundationOne. Areas of interest were circled on the H&E slide (tumor percentage 30%) and corresponding areas from the unstained slides were manually scraped using a razor blade. After deparaffinization with xylene and ethanol wash of the pellet, total nucleic acid was extracted using the RNeasy FFPE Mini Kit (QIAGEN) excluding the DNAase treatment step. The concentration of RNA was determined using Qubit 2.0 fluorimeter (Life Technologies). A relative assessment of the RNA quality was determined using the manufacturer's real-time polymerase chain reaction (PCR) assay for a 113-bp exon junction spanning RNA amplicon from the *VCP* gene, which is included in the Comprehensive Thyroid and Lung (CTL) FusionPlex Assay (ArcherDx). A *C*_t_ value of less than 30 is considered acceptable for subsequent testing.

### Genomic Analysis

Total RNA (250 ng) was reverse-transcribed to cDNA. Libraries were prepared using the CTL FusionPlex Assay for Illumina Platform (ArcherDX) following the manufacturer's protocol (see Zheng et al. 2014 for principle) and sequenced using MiSeq and NextSeq (Illumina). Data was analyzed using the CTL Target Region File and vendor-supplied software (Archer Analysis version 5.0). A minimum of five reads with three or more unique sequencing start sites that cross the breakpoints were set as the cutoff to call for strong evidence of fusions.

## VARIANT INTERPRETATION

Testing at FoundationOne identified an *NRF1-BRAF* fusion and a frameshift variant. No detailed information regarding the breakpoints was provided. A frameshift variant in the *EP300* gene, which was predicted to result in premature termination of protein translation (p.W403fs*29), was also reported.

The *NRF1-BRAF* fusion was subsequently detected using the CTL FusionPlex assay (ArcherDx) validated by our laboratory. Archer Analysis detected that the fusion transcript involved breakpoints at exon 10 of both genes (Table 1; Fig. 1B). There were 173 reads and 15 unique start sites. The resulting *NRF1-BRAF* fusion product joins the amino terminus of the *NRF1* gene with the entire kinase domain of the *BRAF* gene (Fig. 1C). *EP300* was not included in our testing.

**Table 1.** Genomic breakpoints of the *NRF1-BRAF* fusion in our patient by ArcherDx CTL Panel

Gene	Chr	HGVS DNA ref	HGVS protein ref	Variant type	Predicted effect	Allele frequency	Target coverage
*NRF1-BRAF*	7	t(7;7)(q32.1;q34)(hg19 Chr 7: g.129367205_140482957	n/a	*NRF1-BRAF* fusion	Activating/oncogenic	n/a	173

*BRAF* encodes a serine-threonine protein kinase that is highly utilized in the MAP/ERK signaling pathway to drive cell differentiation and division. *BRAF* mutations are commonly implicated in driving oncogenesis in solid tumors and hematopoietic malignancies, which has prompted the development of targeted therapies for the treatment of malignant melanoma, anaplastic thyroid carcinoma, and metastatic non-small-cell lung cancer that harbors *BRAF* p.V600E mutation (Sridhar 2017). *BRAF*-V600E mutated protein functions as a monomer to activate the MEK/ERK signaling pathway. The first-generation BRAF inhibitors vemurafenib and dabrafenib, which directly target BRAF monomeric activity, are effective for treating *BRAF*-V600E-mutated tumors (Janku 2018; Maraka and Janku 2018).

Rearrangements of the *BRAF* gene are rare. Ross et al. (2016) analyzed 20,573 cases of solid tumors and detected *BRAF* rearrangements that contained the entire kinase domain in 55 cases (0.3%). *BRAF*-fusion product, unlike the V600E-mutated protein, functions as a dimer. Inhibition of one of the dimers leads to paradoxical activation of MAPK signaling (Sievert et al. 2013). Extensive studies have been focused on reagents that could overcome paradoxical MAPK activation in *BRAF* fusion–positive tumors. Preclinical studies showed that MEK inhibitors, such as trametinib, could effectively inhibit *BRAF* fusion–mediated activation of MAPK signaling pathway (Jain et al. 2017). Interestingly, both patients in Ross’ cohort who had clinical outcome available responded to MEK inhibitors (Ross et al. 2016). MEK-mediated phosphorylation of ERK could also be inhibited by ERK inhibitor (Nissan et al. 2013), and second-generation RAF inhibitors showed a promising result in selectively inhibiting ERK signaling driven by *BRAF* fusions, as well as V600E and splicing variant (Zhang et al. 2015; Yao et al. 2019). These agents inhibited ERK signaling by specifically disrupting BRAF-containing dimers but sparing RAF function in normal cells (Yao et al. 2019).

*NRF1* (nuclear respiratory factor 1) is a transcription factor that activates the expression of key metabolic genes important for cellular growth, respiration, heme biosynthesis, and neuron overgrowth (https://www.genecards.org/cgi-bin/carddisp.pl?gene=NRF1). The *NRF1-BRAF* fusion in our patient's tumor contains exon 1–10 of the *NRF1* gene and retains the entire kinase domain of the *BRAF* gene. The only *NRF1-BRAF* fusion–positive case reported in the literature is an anaplastic pleomorphic xanthoastrocytoma (APX) that has breakpoints in exon 5 of *NRF1* and exon 9 of *BRAF* (Phillips et al. 2016). Although this particular fusion with novel breakpoints has not been functionally characterized, several other *BRAF* fusions were shown to be activating and oncogenic (Ciampi et al. 2005; Jones et al. 2008; Palanisamy et al. 2010; Botton et al. 2013; Ross et al. 2016). Immunohistochemistry studies performed in the APX case showed that the *NRF1-BRAF* fusion led to activation of the MAPK pathway (Phillips et al. 2016). In addition, in vivo studies have shown that *BRAF* fusion–transformed cells were sensitive to RAF inhibitors (Palanisamy et al. 2010).

*EP300* encodes the p300 transcriptional coactivator protein, which functions as a histone acetylase via chromatin remodeling and is important for cell proliferation and differentiation (https://www.genecards.org/cgi-bin/carddisp.pl?gene=EP300). Mutations in *EP300* have been identified in multiple tumor types including UC (COSMIC database). Studies of truncating *EP300* mutations suggested that it functioned as a tumor suppressor (Gayther et al. 2000). In melanoma, increased BRAF and cytoplasmic p300 expression was reported to be correlated with disease progression (Bhandaru et al. 2014).

Urothelial carcinoma accounts for 90% of bladder cancers, and 25% of patients have muscle-invasive or metastatic disease at the time of presentation (Robertson et al. 2017). Current treatment relies on surgery and chemotherapy. For patients who progressed during neoadjuvant therapy, checkpoint inhibitors could be added (Bellmunt 2019). Recent discoveries using NGS have revealed genomic heterogeneity of UC (Glaser et al. 2017). A comprehensive study by Robertson et al. (2017) analyzed a complete TCGA cohort of 412 muscle-invasive UCs using multiple platforms. Similar to lung adenocarcinoma, melanoma, and squamous cell carcinoma of the head and neck, UC has a high mutation burden that is mainly driven by APOBEC-mediated mutagenesis. The most commonly mutated genes were *KMT2C*, *ATM*, *FAT1*, *CREBBP*, *ERBB2*, *SPANT1*, and *KMT2A*. RNA-seq identified 784 gene fusions but no *BRAF* fusion was found. RNA and miRNA expression clustered the muscle-invasive UC into five subtypes with different survivals. The identification of these genomic aberrations opens the door for the development of targeted therapies.

Upper urinary tract urothelial carcinoma (UTUC) accounts for only 5%–10% of urothelial malignancies, but 60% of UTUCs are invasive at the time of diagnosis, despite increasing awareness and surveillance in the medical community (Munoz and Ellison 2000; Sridhar 2017). Although histologically similar to urothelial bladder carcinoma, UTUC has unique molecular features with mutations in *FGFR3*, *KMT2D*, *PIK3CA*, *TP53*, and *APOBEC* being the most common mutational signature (Thoma 2017) and genetic predisposition for patients with Lynch syndrome (Moss et al. 2017; Audenet et al. 2018). Because of the rarity of this tumor and lack of knowledge of its genomic aberrations, current cancer treatment guidelines in the United States simply include it as a subtype of UC with no specific targeted therapies.

## SUMMARY

*BRAF* fusion has never been reported in UC including UTUC. This is the first report of an *NRF1-BRAF* fusion with novel breakpoints in a metastatic UC of renal pelvic origin. The apparent response of the patient's metastatic disease to trametinib suggests there is utility for treatment of *BRAF* fusion–positive tumors with MEK inhibitors, especially for those patients who have failed initial options.

## ADDITIONAL INFORMATION

### Data Deposition and Access

The genomic variant for our patient case was deposited into ClinVar (https://www.ncbi.nlm.nih.gov/clinvar/) and can be found under accession number SCV000864217.

### Ethics Statement

The patient was deceased. No institutional review board (IRB) review or consent is required at our institution for a single case report on a de-identified patient or deceased patient. All authors declare no conflict of interest.

### Author Contributions

A.L.I. acquired clinical data and images, performed literature search, and drafted the report. N.V.G. analyzed the data, prepared figures, and edited the report. A.D.B. was responsible for the performance of the in-house assay, provided critical review, and edited the manuscript. D.M. interpreted the molecular findings, wrote the manuscript, and guided the project.

### Acknowledgments

The authors would like to acknowledge Ms. Patricia Gereau for assistance with editing the report.

### Funding

The work had no specific funding.

### Competing Interest Statement

The authors have declared no competing interest.

### Referees

Payal Jain

Vivek Subbiah

Anonymous
